# Morphine Sulphate

**Published:** 1897-03

**Authors:** W. H. Phillips


### MORPHINE-SULPHATE.

Dull and stupid; mind seems to wander, forgets what he is sent to do. Answers questions very slowly. Disinclination to do anything. Sleepy all the time, goes to sleep while eating; while talking to him vertigo at times; all things seem to turn in a circle, especially to the left. Great dryness of the mouth. Nose obstructed, breathes through it with difficulty. Blows clots of bright red blood from nose.

Pupils sluggish and very much contracted.

Much difficulty in swallowing, at times almost to strangulation. Oppressed anxious breathing. Urinates with difficulty, passed easier while sitting. Urine scanty. Urine dark, with dark sediment. Alternate constipation and diarrhœa; when constipated stool hard, black, and in little balls. Diarrhœa thin, black, watery stools; sometimes involuntary. Sudden desire for stool, must go quick or is passed immediately. Involuntary stool when he tries to pass urine.

Fingers feel cold with blueness under nails.

Limbs from knees down feel like lead, especially upon moving. Twitching of the lower limbs, cramps in the calves of the legs.

Dryness of the skin. Chilly, wants to be covered, even in a warm room.

